# The International Cotton Genome Initiative workshop I, at PAG XIII

**DOI:** 10.1002/cfg.470

**Published:** 2005-04

**Authors:** R. J. Kohel, John Z. Yu

**Affiliations:** Crop Germplasm Research Unit Southern Plains Agricultural Research Center ARS USDA, 2765 F&B Road College Station TX 77845 USA


**Comparative and Functional Genomics**
*Comp Funct Genom* 2005; **6**: 170–173.
Published online in Wiley InterScience (www.interscience.wiley.com). **DOI:** 10.1002/cfg.470

**Conference Paper**

### The International Cotton Genome Initiative Workshop I, at PAG XIII[†]

R. J. Kohel* and John Z. Yu
*Crop Germplasm Research Unit, Southern Plains Agricultural Research Center, ARS, USDA, 2765 F&B Road, College Station, TX 77845, USA*

**Correspondence to:*
*R. J. Kohel, Crop Germplasm Research Unit, Southern Plains Agricultural Research Center, ARS, USDA, 2765 F&B Road, College Station, TX 77 845, USA.*
*E-mail: kohel@qutun.tamu.edu*

[†] *This article is a U.S. Government work and is in the public domain in the U.S.A.*

Received: 24 January 2005
Accepted: 7 February 2005

The International Cotton Genome Initiative (ICGI) Workshop I was held at Plant and Animal Genomes (PAG) XIII on Sunday 16 January 2005. ICGI has held two biennial meetings at which the membership has had the opportunity for scientific exchange, the first at Nanjing, China, in 2002, the second at Hyderabad, India, in 2004, and the next will be held in 2006 (host country to be selected). The ICGI workshop at PAG XIII offered the unique opportunity to interact and draw upon the resources of the wider genomics community. As the first official PAG-ICGI workshop, and as a fledging organization, it was considered appropriate to review the origins of ICGI and what its future should be. In this process, we wanted to draw upon the experience of other crops with regard to how they have established and maintained their cooperation and how they have gone about establishing their goals. We also wanted to draw upon the current activities of ICGI to evaluate its role in international exchanges. From its beginnings, ICGI recognized the need to be inclusive and has encompassed activities from germplasm resources to the delivery of information and materials to the user community. The following six topics were discussed: (1) why and how ICGI came about; (2) international models of cooperation in other crops; (3) how other crops have established their genomic activities; (4) the role ICGI has played in international interactions; (5) the role ICGI has played in interactions with the private sector; and (6) what is the role and scope to which ICGI should strive? The complete presentations will be posted on the ICGI website; **http://icgi.tamu.edu**.

'The International Cotton Genome Initiative (ICGI): how did we get here and why?' was presented by **Curt L. Brubaker**, from the Centre for Plant Biodiversity Research, CSIRO Plant Industry, Canberra, Australia. In February 2000, a small group of cotton scientists met in Canberra, Australia, to discuss why molecular markers were not being used effectively in cotton breeding. It was agreed that the limited application of molecular markers in cotton breeding was symptomatic of a broader issue. Cotton genomics, in general, had lagged behind other crops. The impressive gains achieved in *Arabidopsis*, rice, wheat and mammalian genomics demonstrated that the practical and analytical tools were at hand to progress cotton genomics to a level that would produce novel evolutionary, structural and functional insights. However, the perception that the uptake of new technologies in cotton research was sluggish was not fair. New technologies were being incorporated into cotton genomics research but, unlike other crops, the cotton genomics community was not realizing the benefits of coordinated communication and collaboration.

The solution was clear. An organization was needed that could bring the international cotton genomics community together to improve communication and collaboration: thus the International Cotton Genome Initiative (ICGI). An interim steering committee was established, and the objective and goals of ICGI presented to the research community (*J Cotton Sci* 2000; **4**:149–151). The private community supported the development of ICGI and contributed funding for participation in meetings. An organizational workshop was held in 2001 in Montpellier, France. An interim steering committee and five workgroups were established; Structural Genomics; Functional Genomics; Germplasm and Genetic Stocks; Evolutionary and Comparative Genomics; and Bioinformatics. Biennial meetings were established for scientific exchanges among the membership, the first at Nanjing, China, in 2002, the second at Hyderabad, India, in 2004, and the next to be scheduled in 2006. A permanent steering committee was established, elected by the membership, which consists of the steering committee chair and chair-elect, and the chair and chair-elect from each of the workgroups. From the original meeting of a dozen individuals, ICGI now has over 440 members from 33 countries. ICGI remains committed to its mission:

> To increase knowledge of the structure and function of the cotton genome for the benefit of the global community and to facilitate: global communication, collaboration, and education; knowledge and resource integration; technology and resource development; and coordinated research planning.

'The International Triticeae Mapping Initiative: 15 years of evolution' was presented by **Mark E. Sorrells** of Cornell University, Ithaca, NY. The talk was co-authored by **J. Perry Gustafson** of the USDA-ARS Plant Genetics Research Unit, Department of Agronomy, University of Missouri, Columbia, and **Patrick E. McGuire** and **Calvin O. Qualset** of the Genetic Resources Conservation Program, University of California, Davis. The international model of cooperation in another crop was the well established and long standing International Triticeae Mapping Initiative (ITMI). ITMI was established in 1989 by a group of researchers interested in developing linkage maps for species in the tribe Triticeae of the family Poaceae. Comparative mapping of genomes of diploids and polyploids of wheat and its relatives has been a focal point for ITMI because of the role of diploids in polyploid evolution. ITMI established an office that has overall responsibility for management and administration, which rotates every 4 years. However, the informal nature of ITMI encouraged researchers to share information and materials freely for the common good, which resulted in rapid accumulation of knowledge of evolutionary relationships and genome structure of wheat. Initially, ITMI had a coordinator for each homologous chromosome group; however, ITMI was reorganized in 2000 to emphasize specialized research topics, each having a coordinator. In 1999 ITMI organized the International Triticeae EST Cooperative, to produce and share ESTs that contributed to a US effort (with NSF support) that produced more than 100 000 wheat ESTs. One of the most important components of ITMI was the close interaction with a central database, the *GrainGenes* database for Triticeae and *Avena. GrainGenes* provided a convenient, accessible repository for information on germplasm, traits, genes and molecular markers as well as a way to coordinate activities of each special interest topic. The original concept of ITMI, which has led to continued success of the organization, was that it established open communications and an exchange of ideas, germplasm and information at the international level, which provided benefits to all participants. ITMI maintains this open communication with two annual workshops: one at the Plant and Animal Genome (PAG) meeting in San Diego, CA, and a second that rotates among countries.

'Defining soybean genomic priorities: separating the wants from the needs' was presented by **Randy C. Shoemaker**, ARS, Ames, IA. Soybean genomics is undergoing a priority and goal-setting process similar to the one ICGI is undertaking for cotton genomics. This presentation focused on the activities of the US soybean community. The first step in the process is characterized by asking a group of researchers to identify the genomic tools and resources their community needs to increase its competitiveness in the funding arena; an extensive list quickly develops. Sorting through that list to identify the shortest route to the greatest gain is hard and requires difficult decisions. Over several years, the soybean research community underwent several iterations of this process through workshops supported by the United Soybean Board, the US Department of Agriculture and, finally, the National Science Foundation. A central role in this process was played by the United Soybean Board, which established scientific executive committees to establish priorities. Each workshop produced more refined and focused white papers and reports. The latter workshop involved Agency administrators and highly respected scientists from other research communities. Gaining broad administrative and scientific support of the process and the goals is critical. In the soybean community a final winnowing and focusing of the action plan and priorities list occurs through a five-person executive committee elected by soybean researchers.

'The role of ICGI in international collaboration and exchanges of genomic resources' was prepared by **Tianzhen Zhang**, of Nanjing Agricultural University, China, and delivered by **Xiao-Ya Chen**, of the Shanghai Institute of Plant Physiology and Ecology, in his absence. This report focused on the long-standing collaboration between Nanjing Agricultural University and the Crop Germplasm Research Unit, of the Southern Plains Agricultural Research Center, ARS-USDA, USA, and how it has been strengthened and broadened by the establishment of ICGI. Since cotton plays a critical role as a sustainable fibre product and is viewed as the world's most important cash crop, there are great opportunities to be gained from global efforts in DNA marker screening, functional genomics, regeneration and transformation, and genetic stock development for cotton genomic research. Many studies on functional genes have been carried out which use DNA markers, maps and gene transfer procedures. The genetic resources to study functional genomics in *Gossypium* spp. have grown dramatically in recent years. However, various cotton linkage maps were constructed worldwide and much of the genetic marker data exist at various research centres. The ICGI was created at a meeting of cotton researchers in Canberra, Australia, in 2000. Since then, productive workshops have been held in France in 2001, China in 2002 and India in 2004. The objectives of ICGI are to: (a) reduce redundancy of research effort and maximize the rate of progress by providing a forum for international researchers; (b) foster tool development to integrate genetic and physical maps; (c) accelerate development of a consensus cotton linkage map; (d) foster rapid application of new genomic tools to cotton improvement; (e) develop a comprehensive forum for exchange and communication within the cotton scientific community and with the *Arabidopsis* model genome community; and (f) develop standardized nomenclature for DNA markers, maps, etc. This report, based on the collaborative experiences between Nanjing Agricultural University and the Crop Germplasm Research Unit, concluded that, in the past 20 years, the international collaboration and exchanges of genomic resources have been enhanced through the activities of ICGI.

'The need to foster public–private partnerships' was presented by **P. Vidyasagar**, Chairman & Managing Director, Vibha Agrotech Ltd., Hyderabad, India, who addressed public and private interactions in ICGI, bringing his experience of operating a major planting seed company and being an active participant in ICGI to bear. He identified the unique differences between public and private institutions and the challenges in integrating these. Although the progress made by ICGI was recognized, he expressed the frustration that the diverse community feels in reaching for its goals and the disparity with the progress found in other organisms. To this end, he proposed actions to be taken and following the Bermuda Agreement and Bayh-Dole Act as models for public–private interactions. Since its inception in 2000, ICGI has been striving to promote cotton genomics research. The cotton genome is unique and complex and has two diploid subgenomes in a single nucleus. In spite of this complexity, the level of cotton genomic information has surged in GenBank. The number of cotton DNA sequences alone has increased from 19 022 to 29 402 in a period of 5 months (July–December 2004). While the advance in research is evident, the information is scattered, each laboratory hosting their respective proprietary data. The need for a holistic approach to sequence the cotton genome and a centralized cotton database was stressed at the recently concluded 2004 ICGI workshop in India. Public–private partnerships have had success stories in plant genome projects, with Syngenta and Monsanto contributing data to the International Rice Genome Sequence Project (IRGSP), Pioneer, Ceres and Monsanto sharing data with the Maize Genome Project, and 17 private companies in the *Arabidopsis* Genome Initiative (AGI). The importance of partnerships and alliances in genome projects was stressed in this talk. The expectations of private industry, sustainable benefits and comparative advantages of the partnership were underlined. While other plant genome projects are striding ahead, ICGI has yet to set an agenda for collaborative research. A broad scheme of starting points, suggestions and priorities for ICGI was provided. Lessons from the Bermuda Agreement, IRGSP, AGI and other genome projects were reviewed.

'Charting the future was presented by **Roy G. Cantrell**, Cotton Incorporated, Cary, NC, USA, who was the first Chair of ICGI, and is now the Vice President for Agricultural Research at Cotton Incorporated. His perspectives arise from personal experience with ICGI and in the cotton research–production community. The International Cotton Genome Initiative (ICGI) has been successful in establishing a forum for international scientific exchange and discussion of technical issues related to the cotton genome. Strong international collaboration is essential to unravel the complex cotton genome. He pointed out that international collaborations and exchanges are not without difficulties, but that with well-defined goals and organization these can be overcome. Emphasizing what P. Vidyasagar had said, he stated that the development and maintenance of strong relations with the private sector are essential. There are many technologies, such as DNA marker-assisted selection, that are under-utilized in cotton genetic improvement. ICGI provides one of the few technical platforms for scientific collaboration and discussion in the cotton community. The potential of many of the new genomic technologies is starting to be realized in cotton. At some point in the future, a significant amount of the cotton genome will be sequenced. ICGI is the appropriate forum for mounting the strongest possible international scientific effort in this area, based on common goals and mutual benefit. Of paramount importance will be garnering a broad base of financial resources for a coordinated genome project.

This inaugural annual ICGI workshop of the PAG Conference was successful and has received positive comments and feedback. Future PAG–ICGI workshops will report on and discuss specific issues relating to international collaborations and coordination of cotton genomic research.

